# Distribution of Nd^3+^ ions in oxyfluoride glass ceramics

**DOI:** 10.1186/1556-276X-7-275

**Published:** 2012-05-30

**Authors:** Hua Yu, Hui Guo, Ming Zhang, Yan Liu, Min Liu, Li-juan Zhao

**Affiliations:** 1 The Key Laboratory of Weak-Light Nonlinear Photonics, Ministry of Education, School of Physics, Nankai University, Tianjin, 300071, China; 2 Applied Physics School of TEDA, Nankai University, Tianjin, 300457, China

**Keywords:** Nd, Glass ceramics, Nanocrystal, Distribution

## Abstract

It has been an open question whether Nd^3+^ ions are incorporated into the crystalline phase in oxyfluoride glass ceramics or not. Moreover, relative research has indicated that spectra characters display minor differences between before and after heat treatment in oxyfluoride glass compared to similar Er^3+^-, Yb^3+^-, Tm^3+^-, Eu^3+^-, etc.-doped materials. Here, we have studied the distribution of Nd^3+^ ions in oxyfluoride glass ceramics by X-ray diffraction quantitative analysis and found that almost none of the Nd^3+^ ions can be incorporated into the crystalline phase. In order to confirm the rationality of the process, the conventional mathematical calculation and energy-dispersive spectrometry line scanning are employed, which show good consistency. The distribution of Nd^3+^ ions in oxyfluoride glass ceramics reported here is significant for further optical investigations and applications of rare-earth doped oxyfluoride glass ceramics.

## **Background**

Nd^3+^ is always considered to be one of the most efficient rare-earth (RE) ions to generate laser operation around 1.06 μm in different hosts, such as crystals and glasses. In the 1970s, transparent glass ceramics (GCs) with nanocrystals (NCs) of about 50 nm were originally suggested to be used as laser host materials [1,2]. In subsequent oxide glass system studies, the fluorescence lifetimes and absorption spectra of Nd^3+^ ions were measured in neodymium-doped glasses and GCs to investigate the distribution of the Nd^3+^ ions [3]. The results indicated that Nd^3+^ ions were excluded from the crystalline phase and accumulated into the residual glass matrix in the GCs. Also, in a similar glass system, Dymnikov et al. [4] and Kang et al. [5], respectively, found that Nd^3+^ ions exhibited fairly different distribution tendencies when the crystalline phase varied in glass ceramics by performing fluorescence, absorption, and Judd-Ofelt analyses.

In 1993, Wang and Ohwaki, for the first time, reported the fabrication of transparent oxyfluoride GCs which combined the advantages of fluoride glasses with efficient frequency upconversion and oxide glasses with good chemical and mechanical stability [6]. RE ions, specifically Er^3+^ and Yb^3+^ ions, could be dissolved into the crystalline phase with lower phonon energy, generating a remarkable increase of luminescence, which made the transparent GCs potential outstanding laser host materials. Afterwards, extensive studies on RE-doped (Er\Eu\Yb\Ho) oxyfluoride GCs were carried out with various compositions and proportions [7-9]. Nevertheless, fewer studies were performed on Nd^3+^-doped oxyfluoride GCs, different from Er^3+^\Yb^3+^\Tm^3+^\Eu^3+^ ions, as Nd^3+^ ions were found to be difficultly incorporated into the crystalline phase [5,10-12]. Pisarska et al. [10,11] revealed that in oxyfluoroborate glass compositions, the ^4^ F_3/2_ fluorescence lifetime didn't change after thermal treatment, showing that Nd^3+^ ions did not incorporate into the crystalline phase. Abril et al. [13] focused on different preparation methods with various Nd^3+^ ions sources and the corresponding distributions of Nd^3+^ ions in GCs. NdF_3_ was thought to be more helpful for Nd^3+^ ions to be incorporated into the crystalline phase using analysis of fluorescence, absorption, and the Judd-Ofelt theory [13,14]. From the previous research mentioned above, whether in the Nd^3+^-doped oxide glass system, oxyfluoroborate glass compositions, or similar oxyfluoride glass system, Nd^3+^ ions were difficultly incorporated into the crystalline phase, while Wang et al. [15-18] investigated the thermal and optical properties of different kinds of Nd^3+^-doped GCs, which suggested that most of Nd^3+^ ions were doped in the crystalline phase.

While most of the previous research focused on incorporating the RE ions into the nanocrystals, such as Er^3+^\Yb^3+^\Tm^3+^\Eu^3+^ ions, the distribution of Nd^3+^ ions in GCs is somewhat ambiguous and exhibits some peculiar results, which much influences the materials' fluorescence properties. As previously reported about the distribution of Nd^3+^ in various oxyfluoride glass ceramics, indirect characterization techniques, for example, fluorescence analysis, X-ray diffraction (XRD), Judd-Ofelt theory calculation, and the like, were used. The properties of samples doped with Nd^3+^ ions show small changes after thermal treatment. Even with the slight changes caused by the distribution of Nd^3+^ ions in the nanocrystalline phase or glass phase, the Nd^3+^ ion segregation in the glass matrix also leads to the same results, that is to say that most of the results obtained from the indirect characterization technique cannot distinguish between Nd^3+^ ions entering into the nanocrystalline phase and locating on the interface between the crystalline and glassy phase. Nowadays, direct characterization technique energy dispersive X-ray spectroscopy (EDS, conventional fixed point measurement) was used to study the distribution of Nd^3+^ ions in glass ceramics. Since the crystal size is smaller than the probe beam, the analyzed volume in GC always includes both crystalline and glassy phases; the results of EDS do not seem to be entirely reliable.

In this paper, a direct and quantitative investigation on the distribution of Nd^3+^ ions in GCs was performed. Transparent GCs doped with Nd^3+^ ions were prepared, and subsequently, the glass matrix was etched off for releasing the fluoride nanoparticles (NPs) into the water phase for further study. In contrast, investigating the EDS of NPs rather than that of GCs is a more scientific and more convenient method to obtain the chemical composition of NP. The Nd^3+^ ion distribution model was built in GCs and NPs. EDS line scan by high-angle annular dark-field scanning transmission electron microscopy (HAADF-STEM) on the concentrations of concerned elements and the Rietveld full-pattern fitting algorithm were employed for quantitatively clarifying the distribution of Nd^3+^ ions in GCs. The results will be of great significance for the RE distribution investigations and further applications of Nd^3+^-doped glass ceramics.

## **Methods**

Precursor glasses (PGs) with the composition of 44SiO_2_-5Al_2_O_3_-40PbF_2_-10CdF_2_-1NdF_3_ (mole ratio) were fabricated with a traditional melting-quenching route. Transparent GCs were obtained by following thermal treatment at 440 °C under the guidance of differential scanning calorimetry result of as-quenched precursor samples. The GCs were then ground into powder, immersed into hydrofluoric acid solutions, and stirred with a magnetic stirrer so as to thoroughly release the nanocrystals from the glass matrix. Afterwards, nanocrystal powder was obtained by vacuum drying at 80 °C. This fabrication method has been reported in detail in the previous work [19].

All XRD data were obtained with a Rigaku D/Max-2500 diffractometer (Rigaku Corporation, Tokyo, Japan), using CuKα as the radiation. Quantitative analysis of the phases in NCs was carried out with material analysis using diffraction (MAUD) program (Luca Lutterotti, University of Trento, Trento, Italy) [20,21] applying the RITA/RISTA method based on the Rietveld full-pattern fitting algorithm with XRD data in the range of 10° to 125° acquired in step-scan mode with a step of 0.02° (2*θ*) at a counting time of 1 s per step. The program was developed to analyze diffraction spectra and obtain crystal structures, quantity, and microstructure of phases along with the texture and residual stresses. High-resolution transmission electron microscope (HRTEM) analysis was performed to observe the morphology of samples on a Philips TZOST TEM (FEI Co., Hillsboro, OR, USA) operating at 200 kV. STEM and EDS line scan were performed on a Tecnai G2F30 field-emission transmission electron microscope (FE-TEM; FEI Co., Hillsboro, OR, USA) using HAADF as imaging mode. All the measurements proceeded under the same condition.

## **Results and discussion**

**Figure 1:**
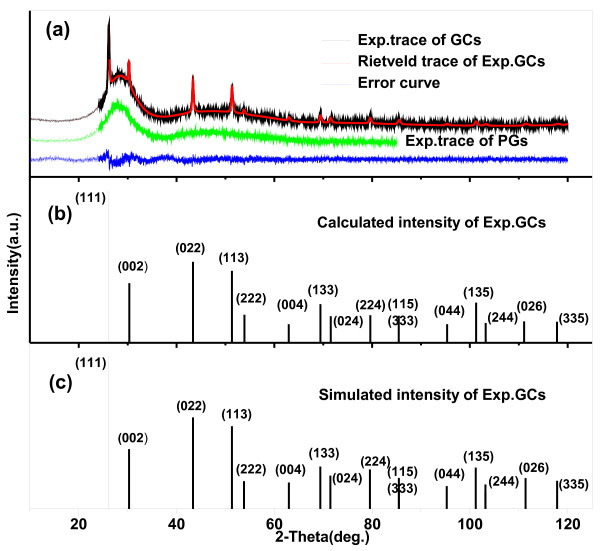
**X-ray diffraction patterns of GCs and PGs.** (**a**) Rietveld analyses for XRD pattern of GCs and the error curve. (**b**) The calculated line spectrum from peak area of GCs and (**c**) the simulated line spectrum of GCs from the Diamond program are presented for comparison.

Figure 1a shows the diffraction patterns of compounds of GCs, along with the diffraction pattern of PGs without thermal treatment. Two curves show an overlap of the peaks corresponding to the amorphous matrix of the Si-Al glass. The differences between PGs and GCs indicate that the crystallization process takes place in the glass matrix under thermal treatment, and the characteristic diffraction peaks of pure cubic β-PbF_2_ (JCPDS: 06–0251) emerge. XRD patterns of the GCs were compared with the theoretical calculation by program Diamond 3.1day (Crystal Impact GbR, Bonn, Germany) based on the structure of pure cubic β-PbF_2_. Since the calculation results can only be expressed in the form of a vertical bar theoretically (see vertical bars in Figure 1c), the XRD trace (raw data) was converted to the form of integral intensities of peaks (i.e., the peak area value, see vertical bars in Figure 1b) in order to compare with the theoretical results. Indexing was also taken as exhibited in Figure 1b,c. As seen in Figure 1b,c, the positions and integral intensities of the peaks derived from the experiment are very consistent with the calculation based on the structure of pure cubic β-PbF_2_. Quantitative calculation indicates that the average relative errors between the theoretical and experimental values are only 0.98% and 4.37% in 2*θ* and the relative intensity for every peak, respectively. In order to further confirm the validity of the nanocrystal structure, pure cubic β-PbF_2_, X-ray data refinement was made using the program MAUD. The analysis was started assuming the structure of β-PbF_2_ (*F*_m3m_ no. 225) for the nanocrystal phase and the structure of cubic amorphous silica-Al glass (*P*_213_ no. 198) for the amorphous phase. The used crystalline structures can all be found in the database of the MAUD program. The corresponding factors *R*_wp_ (4.69%) and *R*_exp_ (4.27%) indicate a relatively good agreement between the experimental and calculated patterns. According to the results of the obtained quantitative analysis, about 8.6 mol% to 13.5 mol% of the original Pb would contribute to form crystalline β-PbF_2_ during the thermal treatment. In addition, the structure cell parameter of the nanocrystal phase corresponds to that of the pure cubic β-PbF_2_, which further checks the structure of the nanocrystal phase.

**Figure 2:**
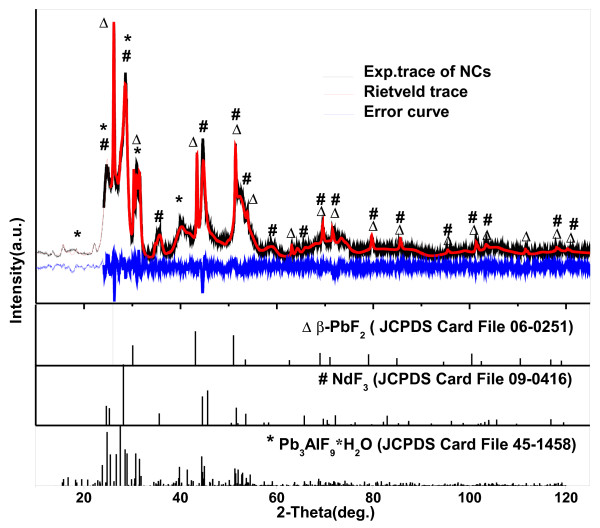
**Rietveld analyses for XRD pattern of NCs and the error curve.** The line spectrum of β-PbF_2_, NdF_3_, and Pb_3_AlF_9_·H_2_O are revealed for comparison.

The Nd^3+^ ions could not be incorporated into the nanocrystal phase by the reason that the structure of the nanocrystal phase is pure cubic β-PbF_2_. Corrosion-treated methods were applied to acquire the NCs so as to explore the information on chemical compositions and structures of nanocrystals and weaken the interference of the glass matrix. For Nd^3+^-doped samples, an unusual change happens when the nanocrystals embedded in the glass matrix abstracted by etching process. Unlike our previous work [22] of Er^3+^/Yb^3+^-codoped GCs wherein only β-PbF_2_ phase was observed, the complex mixture was obtained after etching off the glass matrix of GCs doped with Nd^3+^. With peak indexing, the additional intense diffraction peaks appear to be NdF_3_ (JCPDS: 09–0416) and Pb_3_AlF_9_·H_2_O (JCPDS: 45–1458) phases. The new crystal phase NdF_3_ is generated in the course of the etching treatment, wherein Nd^3+^ ions existing in the glass matrix react with F^−^ ions in acid, generating NdF_3_. Likewise, Pb and Al ions of the glass matrix reacting with F^−^ ions in acid generate the Pb_3_AlF_9_·H_2_O phase. MAUD program based on the Rietveld method was used to calculate the relative amounts of β-PbF_2_ and NdF_3_ phases. The crystal structure information of the concerned phases is acquired from the Crystallography Open Database [23]. The refinement results and difference plot for the observed and calculated patterns of NPs are shown in Figure 2. The refining factors of *R*_wp_ (8.07%) and *R*_exp_ (4.02%) indicate a good agreement between the experimental and calculated patterns. The structure cell parameter of β-PbF_2_ obtained from the refinement of NCs (0.5890 nm) and GCs (0.5898 nm) is nearly the same as that of pure β-PbF_2_ (0.5927 nm), which further verifies the existence of Nd^3+^ outside the NCs. According to other relative results obtained from Rietveld analysis, the cell parameters of the other two phases are nearly the same as those of the standard structures separately, which further certifies the authenticity of the complex phases suggested above. From the results of the relative weight fraction obtained from refinement, moreover, through the sample arithmetic conversion, we can assume that about 10.9 mol% of the Pb of the raw material contribute to form the crystalline β-PbF_2_ phase during the thermal treatment. This conclusion is in accordance with the Rietveld analysis data from Figure 1.

**Figure 3:**
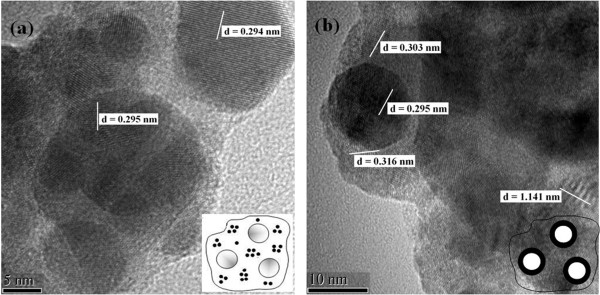
**High-resolution TEM micrographs with marked interplanar distance*d*values of (a) GCs and (b) NCs.** The insets are the simplified models for GCs and NCs, where the large white spheres stand for β-PbF_2_ nanocrystals, and small black spheres in GCs are for Nd^3+^ ions.

High-resolution TEM micrographs of GCs and NCs are displayed in Figure 3, providing a visual characterization of the samples and a direct analysis method. In GCs, as shown in Figure 3a, only spherical β-PbF_2_ nanocrystals with an average size of about 22 nm distribute in the glass matrix, which has a good consistency with the calculated size using the Scherrer formula. However, in NCs, besides the 22-nm nanocrystals already existing in GCs, as shown in Figure 3b, a large amount of additional crystals and flake-like substrates also emerges. Particularly, most of these additional crystals assemble around the known β-PbF_2_ nanocrystals, forming a core-shell-like structure. According to the HRTEM micrographs, the periodic arrayed crystal planes can be viewed clearly, and a part of symbolic interplanar distance (*d*) values, which were measured accurately for both GC and NCs, has been labeled. The measured values of *d*, 0.294 or 0.295 nm for Figure 3a,b, are attributed to the crystal plane (002) of pure cubic β-PbF_2_ as the core, while the *d* values of 0.303 and 0.316 nm around the β-PbF_2_ nanocrystal belong to crystal planes (020) and (−121) of the shell NdF_3_, respectively, and the other one *d* value of 1.141 nm is for the symbolic crystal plane (001) of the substrates Pb_3_AlF_9_·H_2_O. This conclusion is in accordance with the XRD refinement result of NCs, which has been displayed in Figure 2 above. On the basis of Rietveld refining results and HRTEM micrographs, the Nd^3+^ ions are reserved in the glass matrix after thermal treatment and aggregated outside β-PbF_2_ particles in the form of NdF_3_ crystals when the glass matrix is removed through the etching process. Therefore, a simplified model is proposed to describe the distribution of Nd^3+^ ions both in GCs and NCs, as shown in the insets of Figure 3a,b. That is to say, with thermal treatment on PGs, the phase segregation process happens, and on the one hand, as shown in the inset of Figure 3a, Nd^3+^ ions with a nonhomogeneous distribution form an aggregate structure, which has been reported on the previous research [13,14]; on the other hand, Pb and F elements get together to form high-temperature phase β-PbF_2_ nanocrystals, while the formation of Nd^3+^ clusters prevents the process of incorporation into the β-PbF_2_ phase. During the etching process for removing the glass matrix, the Nd^3+^ ions meet F^−^ ions in the acid solution and generate NdF_3_ crystals. Then, the large quantities of NdF_3_ crystals adsorb the surface of the β-PbF_2_ nanoparticles. The model for NCs is simplified as a core-shell structure for further analysis, *viz.*, the β-PbF_2_ particle as the core and the covering NdF_3_ crystals as the shell, as shown in the inset of Figure 3b. This rough simplification is acceptable considering the quantitative analysis result of NCs.

**Figure 4:**
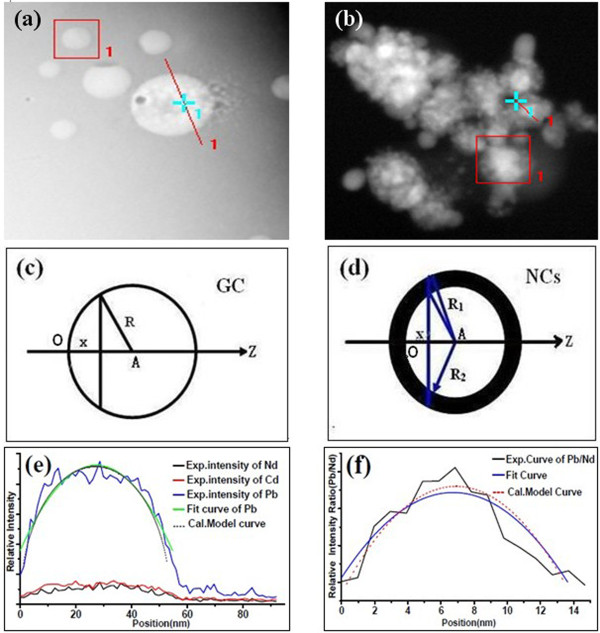
**EDS line scan graphs in HAADF mode (a,b).** EDS line scans for the ideal models of (**c**) crystalline β-PbF_2_ and (**d**) core-shell structure in NCs. (**e**) The relative element signal intensity of Pb, Nd, and Cd in GC; the fit curve; and the calculated model curve of element Pb are presented for comparison; (**f**) The fit curve of relative element intensity ratio Pb/Nd in NCs and the calculated core-shell model curve are also shown.

In order to verify the anticipation, EDS line scans in HAADF mode across the β-PbF_2_ particles are carried out on a Tecnai G2F30 FE-TEM to offer signal intensity values for different elements. The line scanning paths are shown in Figure 4a,b, and the results are shown in Figure 4e,f. In GCs, the detected trace of Nd in EDS like that of Cd indicates that the nanocrystalline phase is pure β-PbF_2_, rather than β-PbF_2_:RE, as previously suggested by many researchers. Even the trace of Nd in EDS of the other samples doped with different concentrations of Nd, the former researches confirmed that Cd hardly congregated in the nanocrystals, which also indicated that none of the Nd ions were incorporated into the β-PbF_2_ crystalline phase [22,24,25]. Figure 4c shows the EDS line scan for the ideal spheric body model of crystalline β-PbF_2_ in GCs. As an ideal sphere model, dot *A* is for the center of it. Axial direction (*Z*) is the orientation of the EDS line scan. Dot *O* and length *x* are the initial point and the distance of the line scan separately. From Figure 4c, we can get that from the relation formula, the content of Pb satisfies

(1) $$Y_{1}\propto 2\sqrt{R^{2}-{\left(R-x\right)}^{2}}$$

where *Y*_1_ is the relative intensity of element Pb, *R* is the half size of the β-PbF_2_ nanocrystal, and *x* is the length of the line scan. Through conventional mathematical analysis, the simulated curve is shown in Figure 4e, which is in accordance with the fitting curve of the experimental measurement. In the same way, the ideal core-shell structure model of NCs, which is shown in Figure 4d, is analyzed by conventional mathematical simulation, as shown in Figure 4f. From Figure 4d, the signal intensity ratio of the content of Pb and Nd can be expressed by the following formula:

(2) $$Y_{2}\propto \frac{\sqrt{R_{2}^{2}-{\left(R_{2}-x\right)}^{2}}}{\sqrt{R_{1}^{2}-{\left(R_{2}-x\right)}^{2}}-\sqrt{R_{2}^{2}-{\left(R_{2}-x\right)}^{2}}}$$

where *Y*_2_ is the relative element intensity ratio Pb/Nd, *R*_1_ and *R*_2_ are the half sizes of the core-shell model and β-PbF_2_ nanocrystal respectively, and *x* is the length of the line scan. The accordance with experimental results, as shown in Figure 4e,f, proves the rationality of the model. Therefore, it is concluded that all of the Nd^3+^ ions are located in the glass matrix, while few of the Nd^3+^ ions dope into crystalline phase.

As a result from that, due to Nd^3+^ ions aggregating into the glass matrix rather than incorporating into the β-PbF_2_ crystalline phase during thermal treatment, Nd^3+^ ion-doped oxyfluoride glass exhibits a weak difference in fluorescence and other optical properties before and after heat treatment. On one hand, the location of Nd^3+^ ions is the oxide environment with a higher coupled phonon energy, which will boost nonradiative relaxation process, compared with fluoride nanocrystal phase; on the other hand, aggregation of Nd^3+^ ions can also bring some much smaller effects on fluorescence and optical properties than that of GCs doped with Nd^3+^ ions into the fluoride nanocrystal phase. This is in agreement with some previous research, such as what was discussed by Rapp et al. in [3]; the fluorescence lifetimes of Nd^3+^ ions were much shorter than those in glasses, and the absorption and emission spectra of Nd^3+^ ions were identical both in neodymium-doped glasses and GCs, which indicated that neodymium ions were excluded from the crystalline phase of GCs and were entirely accumulated in the glass matrix. However, the most noteworthy thing is that the segregation of Nd^3+^ ions in the glass matrix also leads to the same results; in other words, most of the results obtained from indirect characterization techniques cannot distinguish between Nd^3+^ ions entering into the crystalline phase and locating on the interface between the crystalline and glassy phase. Therefore, unlike those indirect characterization techniques used by most of previous research, a direct and quantitative investigation method was employed to present a clear result that almost none of the Nd^3+^ ions can be incorporated into the crystalline phase but reside in the glass matrix.

## **Conclusions**

Transparent Nd^3+^-doped GCs were prepared and corrosion-treated to release NCs from the glass matrix for a direct study on their composition and structure information. Unlike the former results, β-PbF_2_:RE crystalline phase was obtained, and pure β-PbF_2_ crystalline phase was generated after thermal treatment. Especially after etching off the glass matrix, massive NdF_3_ crystals simultaneously generate in free NCs. Through the XRD Rietveld refining method, almost the whole of Nd^3+^ ions reside in the glass matrix despite that the samples undergo the thermal treatment. For further demonstration, the models of Nd^3+^ ions existing in the glass matrix in GCs and the core-shell-like structure of pure β-PbF_2_ surface absorbing NdF_3_ crystals in NCs were built. Then, HRTEM, EDS line scan in HAADF mode, and conventional mathematical analysis were used to verify the models' rationality. The results of experimental characterization well coincide with those of the simulation. Our work explains the previous arguments about whether Nd^3+^ ions doped into the crystalline phase or not and removes the puzzle about minor differences on spectral properties. The study has paved a way to more comprehensively understand the properties of the Nd^3+^-doped oxyfluoride glass. The results here would benefit further research on the optical properties and applications of such materials.

## **Competing interests**

The authors declare that they have no competing interests.

## **Authors' contributions**

LjZ and HY conceived the total study and wrote the manuscript. HG and MZ were involved in the experiments and Rietveld analysis. YL and ML participated in the analysis of HRTEM and EDS. All authors read and approved the final manuscript.

## **Authors' information**

LjZ is a professor in the School of Physics and TEDA Applied Physics School of Nankai University. HY is an associate professor in the School of Physics. HG, MZ, and YL are master students. ML is a doctoral candidate.
